# Supplementary figures and images for: An induced pluripotent stem cell-based chemical genetic approach for studying spinal muscular atrophy
Source: bioRxiv. 2025 Nov 5:2025.11.04.686319. Preprint. [Version 1] doi: 10.1101/2025.11.04.686319 (PMC12637412; doi:10.1101/2025.11.04.686319)

Supplemental Figure 1. L1000 probe pool validation

a

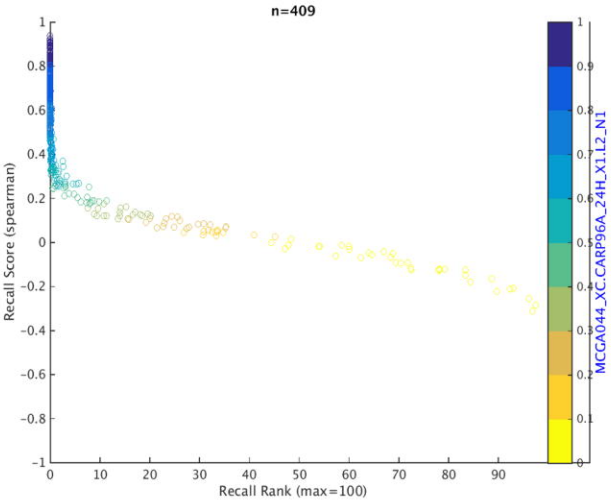

b

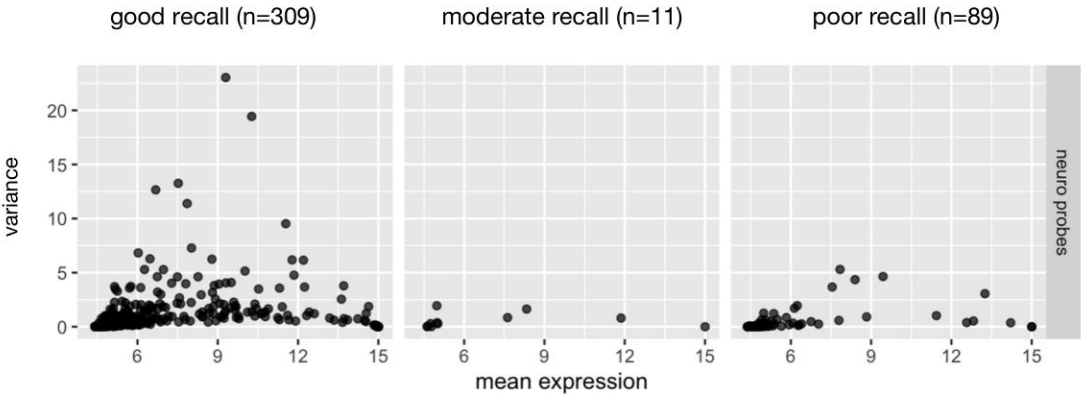

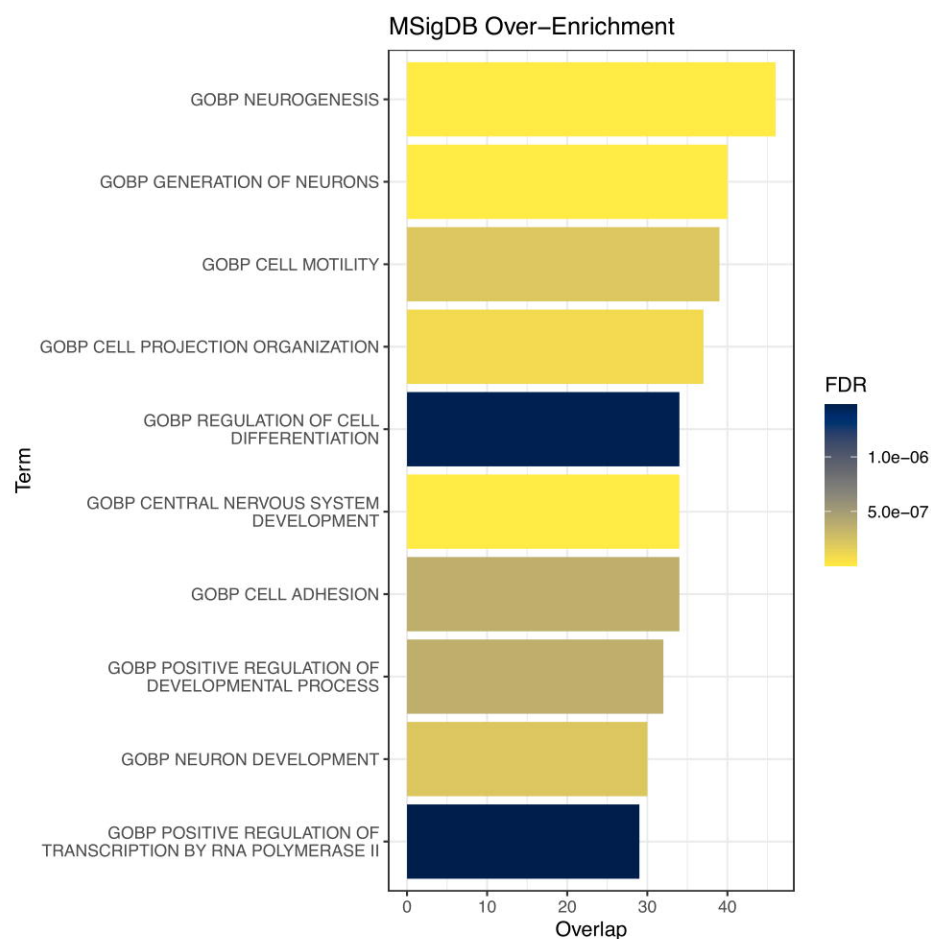

Supplement: 5 — Supplemental Figure 1. L1000neuro probe pool validation. (A) Scatter plot depicting the Spearman correlation vs. the corresponding recall percentile rank for each of the 409 common genes. (B) Scatter plots of the 409 genes’ variance vs. mean expression, derived from the 96 CCLE cell lines’ log2 RPKM values, and stratified by whether the gene had good, moderate, or poor recall, corresponding to recall percentile ranks ≤ 5%, > 5% & ≤ 10%, and > 10%, respectively. Supplemental Figure 2. Pathway analysis of DEGs from type 0/1 and type 3 derived NGN2 neurons. MSigDB over enrichment analysis of DEGs (N=383, p-value < 0.05) from type 0/1 and type 3 SMA iPSC-derived NGN2 cells (see also Supplemental Data File 1). Overlap notes number of genes within each pathway noted. [file NIHPP2025.11.04.686319v1-supplement-5.pdf]
